# Association of XPD Lys751Gln polymorphism with head and neck cancer susceptibility: evidence from 11,443 subjects

**DOI:** 10.1186/1746-1596-9-15

**Published:** 2014-01-20

**Authors:** Hai Lin, Dong Lin, Chunquan Zheng

**Affiliations:** 1 Department of Otorhinolaryngology, Eye and ENT Hospital of Fudan University, 83 Fenyang Road, Shanghai, Xuhui District 200031, China; 2 Department of Biology and Chemical Engineering, Fuqing Branch of Fujian Normal University, Fuqing, Fujian 350300, China

**Keywords:** XPD, Polymorphism, Meta-analysis, Head and neck cancer

## Abstract

**Background:**

Whether the single nucleotide polymorphism (SNP) Lys751Gln of xeroderma pigmentosum group D(XPD) gene increases susceptibility to head and neck cancer (HNC) is controversial and undetermined. Therefore, we conducted this meta-analysis to systematically assess the possible association between them.

**Methods:**

The OVID, Medline, Embase, Pubmed, Web of Science databases were searched to identify the eligible studies. The odds ratio (OR) with 95% confidence interval (95% CI) were used to assess the strength of association.

**Results:**

A total of 11,443 subjects from eighteen studies were subjected to meta-analysis. Overall, XPD Lys751Gln polymorphism had no association with increased HNC risk under all five genetic models (P > 0.05). In the subgroup analysis by ethnicity and source of controls, still no significant association was found under five genetic models (P > 0.05). In the subgroup analysis by cancer type, XPD Lys751Gln polymorphism had statistically significant association with elevated laryngeal cancer (LC) and nasopharyngeal cancer (NPC) risk under heterozygous comparison and dominant model (P<0.05) and borderline significantly increased risk was found under allele contrast for LC and NPC. Carriers of Lys allele and Lys/Lys genotype may be associated with elevated LC and NPC risk.

**Conclusions:**

There is overall lack of association between XPD Lys751Gln polymorphism and HNC risk under all five genetic models and still no significant association was found in the subgroup analysis by ethnicity and source of controls. However, XPD Lys751Gln polymorphism was significantly associated with susceptibility to LC and NPC and the Lys allele and Lys/Lys genotype of XPD Lys751Gln polymorphism may be a risk factor for LC and NPC. However, relatively modest sample sizes were included in this meta-analysis and studies with large sample sizes and representative population are warranted to further clarify this finding.

**Virtual slides:**

The virtual slide(s) for this article can be found here: http://www.diagnosticpathology.diagnomx.eu/vs/5628716106316015.

## Introduction

Head and neck cancers (HNC) which involve malignant neoplasms of the oral cavity, pharynx, and larynx, are the sixth most common cancers threatening human life worldwide [1]. To date, there are ample evidences indicating that HNC is a complex multifactorial disorder involving genetic factors, lifestyle, tobacco smoke, alcohol consuming, and environmental factors [2-6] and some low-penetrant genes have been identified as potential HNC susceptibility genes [7-9]. Among them, an important one is xeroderma pigmentosum group D(XPD) gene, which is located on chromosome 19q13.3. XPD gene, also known as excision repair cross-complementing group 2 (ERCC2) gene, encodes XPD protein, one ATP-dependent helicase within the multi subunit transcription repair factor complex,TFIIH, participates in DNA unwinding during the nucleotide excision repair (NER) pathway and plays a pivotal role in the recognition and repairment of structurally unrelated DNA lesions including bulky adducts and thymidine dimmers [10-12]. Dysregulation of DNA repair proteins in NER pathways may be involved in pathogenesis of cancers [13,14].

The XPD Lys751Gln polymorphism (A35931C, rs13181 or rs1052559) is caused by A to C transition at codon 751 in exon 23 of XPD gene resulting in the Gln substitution for Lys. The XPD Lys751Gln polymorphism may lead to reduction in helicase activity and DNA repair capacity and may be important in the carcinogenesis and development of HNC [15,16].

To date, a series of case–control studies have been conducted to clarify the association between XPD Lys751Gln polymorphism and HNC risk. However, the results were inconsistent. Therefore, we performed this meta-analysis in order to precisely assess the possible association of XPD Lys751Gln with the susceptibility to develop HNC.

## Materials and methods

### Search strategy

The OVID, Medline, Embase,Pubmed, Web of Science databases (up to July 2013) were searched to identify the studies focusing on the association between XPD Lys751Gln polymorphism and susceptibility to HNC. The formats of search terms were used as follows: “xeroderma pigmentosum group D”, “XPD”, “excision repair cross-complementing group 2”, “ERCC2”, “head and neck cancer”, “oral cancer”, “pharyngeal cancer”, “oropharyngeal cancer”, “nasopharyngeal cancer”, “laryngeal cancer”, “SNP or polymorphism or variant” and the combination of them. The literature retrieval was performed by two authors (H. Lin and D. Lin) independently. Relevant reviews and abstracts of meetings were searched for related studies.

### Inclusion and exclusion criteria

Eligible studies which satisfied the following inclusion criteria would be included: 1) the study clearly assessed the association between XPD Lys751Gln polymorphism and HNC risk; 2) HNC was diagnosed by histopathological examination; 3) the normal healthy controls had no diagnosis of HNC. On the other hand, the exclusion criteria was used as follows: 1) studies without normal healthy controls; 2) studies without essential data and information; 3) studies in which the genotype distributions in the controls significantly deviated from Hardy–Weinberg equilibrium (HWE).

### Data extraction

Two authors (H. Lin and D. Lin) performed the extraction of relevant data respectively from all eligible studies. Disagreement was resolved by discussing between two authors (H. Lin and D. Lin). The relevant data as listed below were extracted: name of first author, publication year, country, ethnicity, source of controls, genotyping method, cancer type, total number of cancer patients and controls, and distribution of genotypes in these two groups and P-value of HWE tested in controls. The categorization of ethnicity comprised Caucasian and Asian. Source of controls was categorized as population-based study, hospital-based study, nested case–control study and mixed study. The categorization of cancer type involved laryngeal cancer, oral cancer and nasopharyngeal cancer.

### Statistical analysis

Pooled Odds ratios (ORs) with 95% confidence intervals (CIs) were used to evaluate the association between XPD Lys751Gln polymorphism and susceptibility to HNC on the basis of the distinct genotype and allele frequencies of XPD Lys751Gln polymorphism in two groups. The five distinct genetic models comprised allele contrast (Gln v Lys), homozygous comparison (Gln/Gln v Lys/Lys), heterozygous comparison (Lys/Gln v Lys/Lys), dominant model (Lys/Gln + Gln/Gln v Lys/Lys) and recessive model (Gln/Gln v Lys/Gln + Lys/Lys). We used I^2^ statistic to check heterogeneity. P-value of heterogeneity less than 0.1 was confirmed as statistically significant. The summary ORs were calculated under fixed effects model in the case that P-value of heterogeneity was more than 0.1. Otherwise,we used random effects model to perform the data calculation. HWE in controls was assessed by the online program (http://ihg.gsf.de/cgi-bin/hw/hwa1.pl). Funnel plots,Begg’s test and Egger’s linear regression method were used to evaluate publication bias. P < 0.05 was confirmed as statistically significant to evaluate the data except heterogeneity test. We conducted subgroup analyses by stratification of ethnicity, source of controls and cancer type.In addition, sensitivity analysis was conducted to verify the impact of individual study respectively. All the data statistics and analyses were conducted using Stata version 12.0 (Stata Corporation, College Station, TX).

## Results

### Study characteristics

**Figure 1:**
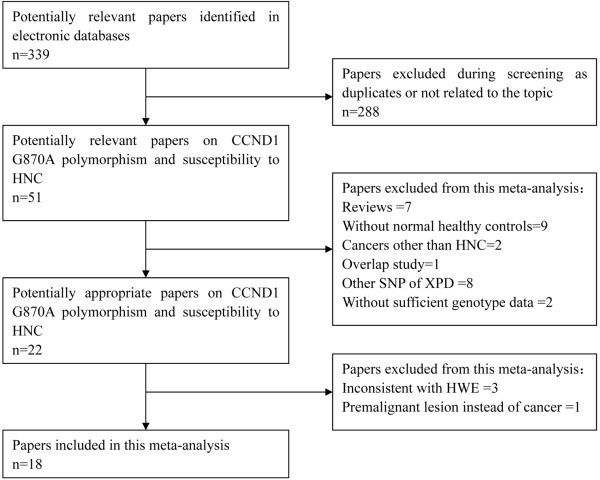
The flow diagram of included/excluded studies.

Selection process was summarized by the flow diagram in Figure 1. In summary, a total of 339 potentially relevant papers were identified after searching the OVID, Medline, Embase,Pubmed, Web of Science databases. Two authors (H. Lin and D. Lin) excluded ineligible articles independently. Then, 288 including duplicates or not related articles were excluded during screening. Then, fifty-one potentially relevant papers on XPD Lys751Gln polymorphism and susceptibility to HNC were selected. After careful examination of these papers, twenty-nine papers were excluded for the following reasons: seven were reviews, nine without normal healthy controls, two on cancers other than HNC, one was overlapped study, eight on other SNP of XPD, two without sufficient genotype data. Then, twenty-two potentially appropriate papers reported the association of XPD Lys751Gln polymorphism with the risk of HNC. However, three papers [17-19] were excluded due to the genotype frequencies of control group being inconsistent with HWE and one paper [20] was ruled out for the study was focusing on premalignant lesion instead of cancer. As a result,eighteen eligible studies [21-38] with a total sample size of 4,510 HNC patients and 6,933 controls were included. In those included studies, eleven studies [21-26,33-36,38] were performed in Caucasians and seven [27-32,37] were conducted in Asians. Thirteen studies [21,22,24,26-28,31,33-38] were hospital-based, three studies [29,30,32] were population-based and one [25] was nested case–control study and one study [23] conducted by Huang et al. was a pooled analysis comprised two population-based studies and one hospital-based study. The study conducted by Huang [23] recruited whites, blacks and others and had overall data of all subjects and white subjects. However, the genotype frequencies of controls in all subjects were inconsistent with HWE, so we only included white subjects. Two studies [21,38] was performed on laryngeal cancer, three studies [27,31,37] on oral cancer, one [29] on nasopharyngeal cancer and twelve studies [22-26,28,30,32-36] on head and neck cancers. We only included six studies [21,27,29,31,37,38] having detailed genotype data of laryngeal cancer, oral cancer and nasopharyngeal cancer for subgroup analysis by cancer type. Consequently, we performed subgroup analysis by stratification of ethnicity, source of controls and cancer type. Details of subjects in these studies were outlined in Table 1.

**Table 1:** Characteristics of included studies

**Author**	**Year**	**Country**	**Ethnicity**	**Source**	**Genotyping method**	**Cancer type**	**Total number**		**Cases**			**Controls**			**HWE of controls**
							**Cases**	**Controls**	**Lys/Lys**	**Lys/Gln**	**Gln/Gln**	**Lys/Lys**	**Lys/Gln**	**Gln/Gln**	
Sturgis [26]	2000	America	Caucasian	HB	PCR-RFLP	SCCHN	189	496	75	83	31	218	221	57	0.9304
Gajecka [21]	2005	Poland	Caucasian	HB	PCR-RFLP	LC	293	320	110	135	48	87	175	58	0.0659
Huang [23]	2005	America	Caucasian	Mix*	TaqMan-PCR	HNC	425	683	176	188	61	296	292	95	0.0951
Rydzanicz [24]	2005	Poland	Caucasian	HB	PCR-RFLP	HNC	172	143	69	73	30	54	64	25	0.4265
Kietthubthew [32]	2006	Thailand	Asian	PB	PCR-RFLP	SCCOC	105	164	83	21	1	126	36	2	0.7489
Matullo [25]	2006	Europe	Caucasian	nest	TaqMan-PCR	UADC	82	1094	34	39	9	397	504	193	0.1330
Ramachandran [37]	2006	India	Asian	HB	PCR-RFLP	OC	110	110	49	46	15	71	31	8	0.0908
An [22]	2007	America	Caucasian	HB	PCR-RFLP	SCCHN	829	854	330	394	105	358	386	110	0.7091
Bau [27]	2007	Taiwan	Asian	HB	PCR-RFLP	OC	154	105	134	18	2	89	15	1	0.6824
Yang [29]	2007	China	Asian	PB	PCR-RFLP	NPC	153	168	128	24	1	124	43	1	0.1805
Majumder [31]	2007	India	Asian	HB	PCR-RFLP	OC	309	388	158	125	26	190	158	40	0.4030
Harth [35]	2008	Germany	Caucasian	HB	PCR-RFLP	SCCHN	312	300	111	154	47	108	149	43	0.4642
Mitra [34]	2009	North India	Caucasian	HB	PCR-RFLP	SCCHN	275	385	88	148	39	163	179	43	0.5571
Jelonek [33]	2010	Poland	Caucasian	HB	PCR-RFLP	HNC	103	110	29	52	22	38	60	12	0.1030
Ji [28]	2010	Korea	Asian	HB	PCR-SBE	SCCHN	267	348	232	32	3	298	48	2	0.9645
Stembalska [38]	2011	Poland	Caucasian	HB	PCR-RFLP	LSCC	60	100	20	26	14	32	46	22	0.4795
Kumar [36]	2012	North India	Caucasian	HB	PCR-RFLP	SCCHN	278	278	92	125	61	129	110	39	0.0531
Yuan [30]	2012	China	Asian	PB	TaqMan-PCR	HNC	394	887	333	57	4	752	129	6	0.8556

*HB* hospital-based study, *PB* population-based study, *two population-based studies and one hospital-based study, *nest* nested case–control study, *PCR-RFLP* polymerase chain reaction-restriction fragment length polymorphism, *PCR-SBE* polymerase chain reaction-single base extension, *HNC* head and neck cancer, *SCCHN* squamous cell cancer of the head and neck, *NPC* nasopharyngeal cancer, *LC* laryngeal cancer, *LSCC* laryngeal squamous cell cancer, *UADC* upper aerodigestive tract cancer, *OC* oral cance, *HWE* Hardy–Weinberg equilibrium.

### Association between XPD Lys751Gln polymorphism and susceptibility to HNC

**Figure 2:**
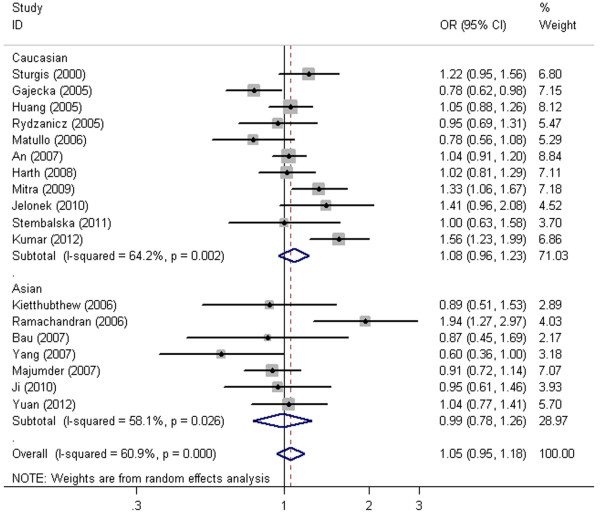
**Forest plot of XPD Lys751Gln polymorphism associated with HNC risk by ethnicity stratification under allele contrast (Gln v Lys).** Random effects model was used.

The main results of our meta-analysis under five distinct genetic models were listed in Table 2. Overall, XPD Lys751Gln polymorphism had no association with increased HNC risk under all five genetic models (allele contrast: OR = 1.05, 95% CI = 0.95-1.18, P = 0.337, Figure 2; homozygous comparison: OR = 1.18, 95% CI = 0.96-1.45, P = 0.118; heterozygous comparison: OR = 1.02, 95% CI = 0.90-1.17, P = 0.725; dominant model: OR =1.05, 95% CI =0.91-1.21; P = 0.538; recessive model: OR = 1.11, 95% CI = 0.98-1.26, P = 0.112).

**Table 2:** Main results of pooled ORs in this meta-analysis

**Study groups**	**n**	**Gln v Lys**		**Gln/Gln v Lys/Lys**		**Lys/Gln v Lys/Lys**		**Lys/Gln + Gln/Gln v Lys/Lys**		**Gln/Gln v Lys/Lys + Lys/Gln**	
		OR (95% CI)	Ph	OR (95% CI)	Ph	OR (95% CI)	Ph	OR (95% CI)	Ph	OR (95% CI)	Ph
All	18	1.05(0.95–1.18)	<0.001	1.18(0.96–1.45)	0.030	1.02(0.90–1.17)	0.011	1.05(0.91–1.21)	0.001	1.11(0.98–1.26)	0.359
Ethnicity	18										
Caucasian	11	1.08(0.96–1.23)	0.002	1.17(0.92–1.49)	0.008	1.06(0.91–1.25)	0.033	1.09(0.92–1.29)	0.004	1.11(0.97–1.27)	0.150
Asian	7	0.99(0.78–1.26)	0.026	1.13(0.75–1.70)	0.437	0.95(0.73–1.23)	0.062	0.97(0.74–1.27)	0.028	1.09(0.74–1.60)	0.687
Source											
HB	13	1.11(0.98–1.27)	0.001	1.26(0.98–1.61)	0.014	1.07(0.91–1.27)	0.009	1.11(0.93–1.33)	0.001	1.16(1.00–1.33)	0.273
Mix	1	1.05(0.88–1.26)	–	1.08(0.74–1.57)	–	1.08(0.83–1.41)	–	1.08(0.85–1.38)	–	1.04(0.73–1.47)	–
PB	3	0.86(0.62–1.19)	0.193	1.25(0.44–3.54)	0.870	0.82(0.57–1.18)	0.181	0.83(0.57–1.19)	0.168	1.26(0.45–3.57)	0.888
nest	1	0.78(0.56–1.08)	–	0.54(0.26–1.16)	–	0.90(0.56–1.46)	–	0.80(0.51–1.27)	–	0.58 (0.28–1.17)	–
Cancer type	6	0.95(0.72–1.25)	0.005	0.88(0.65–1.18)	0.185	0.87(0.60–1.27)	0.007	0.90(0.61–1.31)	0.002	0.97(0.73–1.28)	0.640
LC	2	0.72(0.48–1.10)	0.339	0.72(0.48–1.10)	0.383	0.66(0.48–0.91)	0.347	0.69(0.48–0.97)	0.285	0.93(0.64–1.34)	0.655
OC	3	1.16(0.68–1.98)	0.007	1.08(0.69–1.69)	0.075	1.18(0.67–2.05)	0.036	1.20(0.65–2.21)	0.012	1.02(0.66–1.58)	0.213
NPC	1	0.60(0.36–1.00)	–	0.97(0.06–15.66)	–	0.54(0.31–0.94)	–	0.55(0.32–0.95)	–	1.10(0.07–17.72)	–

*Ph* P-value of Q-test for heterogeneity test, *HB* hospital-based study, *PB* population-based study, *nest* nested case–control study, *LC* laryngeal cancer, *OC* oral cancer, *NPC* nasopharyngeal cancer.

In the subgroup analysis by ethnicity and source of controls, still no significant association was found (Figure 2, Table 2).

**Figure 3:**
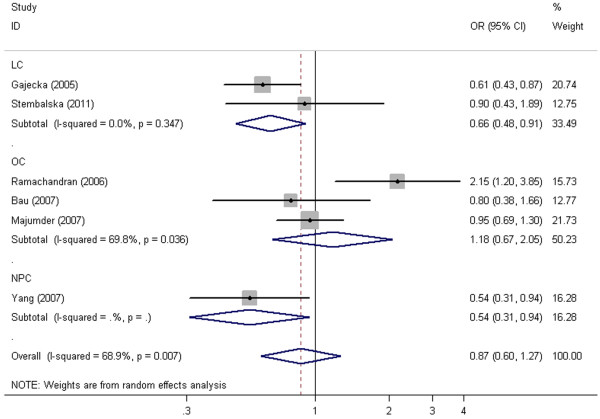
**Forest plot of XPD Lys751Gln polymorphism associated with HNC risk by cancer type stratification under heterozygous comparison (Lys/Gln v Lys/Lys).** Random effects model was used.

In the subgroup analysis by cancer type, XPD Lys751Gln polymorphism had statistically significant association with elevated laryngeal cancer (LC) and nasopharyngeal cancer (NPC) risk under heterozygous comparison and dominant model (P < 0.05, Figure 3, Table 2) and borderline significantly increased risk was found under allele contrast for LC (OR =0.82, 95% CI = 0.67-1.00, P = 0.056) and NPC (OR =0.60, 95% CI =0.36-1.00, P = 0.05). Carriers of Lys allele and Lys/Lys genotype were more likely to have LC or NPC.

### Heterogeneity and publication bias

There were no significant heterogeneities for the overall analysis under recessive model and the subgroup analysis by cancer type under homozygous comparison and recessive model (Ph>0.10, Table 2), so the results were assessed under fixed effects model. However, there were some significant heterogeneities for the overall analysis under allele contrast, homozygous comparison, heterozygous comparison and dominant model and the subgroup analysis by cancer type under allele contrast, heterozygous comparison and dominant model (Ph<0.10, Table 2). Hence, the results were assessed under random effects model.

**Figure 4:**
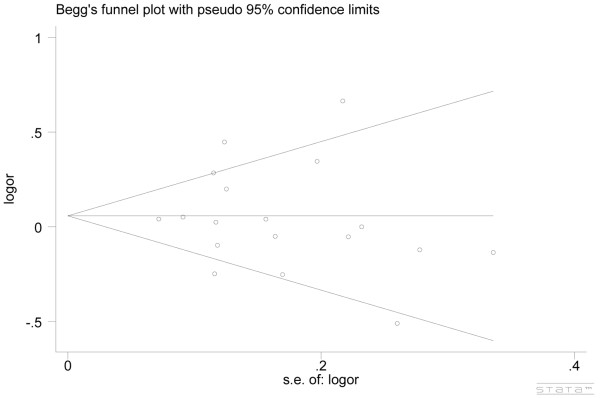
**Begg’s funnel plot with pseudo-95% confidence limits under allele contrast (Gln v Lys).** Each small circle represents a separate study for the indicated association.

In this meta-analysis, we used both funnel plots, Begg’s test and Egger’s linear regression method to evaluate the publication bias. There were no obvious asymmetry in the funnel plots. Meanwhile, results of Begg’s test and Eggers’s linear regression method indicated that there were no obvious publication bias (P > 0.05, Figure 4).

### Sensitivity analysis

Sensitivity analysis was performed to reflect the impact of the individual study to the summarized ORs by removing one study each time involved in the meta-analysis.

**Figure 5:**
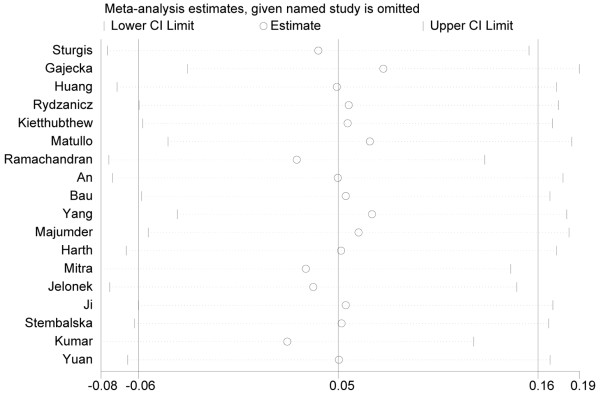
**The result of sensitive analysis under allele contrast (Gln v Lys).** This figure shows the influence of individual studies on the summary OR. The middle vertical axis indicates the overall OR, and the two vertical axes indicate the pooled OR when the left study is omitted from the meta-analysis. The two ends of the dotted lines represent the 95% CI.

We found that the summarized ORs with 95% CIs under all genetic models were not significantly altered after sensitivity analysis (Figure 5), indicating that our results were stable and statistically robust.

## Discussion

It has been shown that XPD acting as a key DNA repair protein in the NER pathway is involved in the pathogenesis of cancer and XPD Lys751Gln polymorphism may be involved in the mechanism of carcinogenesis b [39]. To date, different studies on the association between XPD Lys751Gln polymorphism and HNC risk have showed discrepant results. Thus, our meta-analysis from eighteen studies comprising 4,510 HNC patients and 6,933 controls was performed to precisely assess the possible association of XPD Lys751Gln polymorphism with the susceptibility to develop HNC.

Our meta-analysis, which comprised 4,510 HNC patients and 6,933 controls, indicated the following descriptions: first, XPD Lys751Gln polymorphism had no association with increased HNC risk under all five genetic models by overall analysis; second, still no significant association was found under five genetic models in the subgroup analysis by ethnicity and source of controls;third, XPD Lys751Gln polymorphism had statistically significant association with elevated LC and NPC risk under heterozygous comparison and dominant model and borderline significantly increased risk was found under allele contrast for LC and NPC. The Lys allele and Lys/Lys genotype of XPD Lys751Gln polymorphism may be a risk factor for LC and NPC.

Some limitations of our study should be interpreted. First, the included studies were carried out mainly in Caucasians and Asians and only three studies were population-based, which increased the limitation of statistical power. Hence, studies with larger sample sizes and representative population should be warranted to verify our findings. Second, we only included published papers, as a result, there may be publication bias across studies, although Begg’s test, Egger’s linear regression method did not show any conspicuous publication bias. Finally, our results were grounded on unadjusted estimates, however, XPD Lys751Gln polymorphism is only one phenotype of HNC and HNC is an intricate disorder, and there are many other factors comprising genes, occupation, lifestyle, gender, a history of smoking or drinking, obesity and environmental factors participating in the development of HNC. If the individual data including confounding factors mentioned above were available, a more precise analysis allowing for the adjustment by other covariants should be performed in the future.

## Conclusion

In conclusion, there is overall lack of association between XPD Lys751Gln polymorphism and HNC risk under all five genetic models and still no significant association was found in the subgroup analysis by ethnicity and source of controls. However, XPD Lys751Gln polymorphism was significantly associated with susceptibility to LC and NPC. Carriers of Lys allele and Lys/Lys genotype may be associated with elevated LC and NPC risk. The Lys allele and Lys/Lys genotype of XPD Lys751Gln polymorphism may be a risk factor for LC and NPC. However, relatively modest sample sizes were included in this meta-analysis and studies with large sample sizes and representative population are warranted to further clarify this finding.

## Abbreviations

XPD: Xeroderma pigmentosum group D; SNP: Single nucleotide polymorphism; HNC: Head and neck cancer; LC: Laryngeal cancer; OC: Oral cancer; NPC: Nasopharyngeal cancer; OR: Odds ratio; CI: Confidence interval; HWE: Hardy– Weinberg equilibrium.

## Competing interests

The authors declare that they have no competing interests.

## Authors’ contributions

HL and DL carried out the meta-analysis study, drafted the manuscript and involved in revising the manuscript critically for important intellectual content. CZ and HL participated in the design of the study and revised the manuscript. All authors read and approved the final manuscript.
